# One step from oxides to sustainable bulk alloys

**DOI:** 10.1038/s41586-024-07932-w

**Published:** 2024-09-18

**Authors:** Shaolou Wei, Yan Ma, Dierk Raabe

**Affiliations:** Max Planck Institute for Sustainable Materials, Düsseldorf, Germany

**Keywords:** Materials science, Engineering

## Abstract

Metallurgical production traditionally involves three steps: extracting metals from ores, mixing them into alloys by liquid processing and thermomechanical processing to achieve the desired microstructures^1,2^. This sequential approach, practised since the Bronze Age, reaches its limit today because of the urgent demand for a sustainable economy^2–5^: almost 10% of all greenhouse gas emissions are because of the use of fossil reductants and high-temperature metallurgical processing. Here we present a H_2_-based redox synthesis and compaction approach that reforms traditional alloy-making by merging metal extraction, alloying and thermomechanical processing into one single solid-state operation. We propose a thermodynamically informed guideline and a general kinetic conception to dissolve the classical boundaries between extractive and physical metallurgy, unlocking tremendous sustainable bulk alloy design opportunities. We exemplify this approach for the case of Fe–Ni invar bulk alloys^6,7^, one of the most appealing ferrous materials but the dirtiest to produce: invar shows uniquely low thermal expansion^6,8,9^, enabling key applications spanning from precision instruments to cryogenic components^10–13^. Yet, it is notoriously eco-unfriendly, with Ni causing more than 10 times higher CO_2_ emission than Fe per kilogram production^2,14^, qualifying this alloy class as a perfect demonstrator case. Our sustainable method turns oxides directly into green alloys in bulk forms, with application-worthy properties, all obtained at temperatures far below the bulk melting point, while maintaining a zero CO_2_ footprint.

## Abstract (web summary)

A one-step hydrogen-based redox process turns oxides directly into green alloys in bulk forms, with application-worthy properties.

## Main

**Fig. 1:**
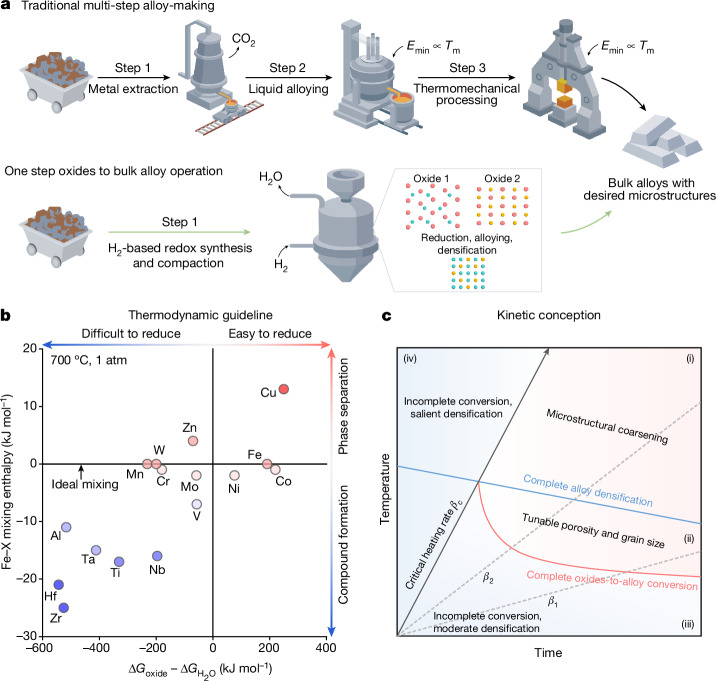
One-step sustainable synthesis of bulk alloys with defined microstructures from oxides. **a**, Schematic comparison between the traditional multi-step alloy-making process and the proposed sustainable ‘one step oxides to bulk alloy’ operation. **b**, Thermodynamically informed design treasure map. For simplicity, here the reducibility ($\Delta {G}_{{\rm{oxide}}}-\Delta {G}_{{{\rm{H}}}_{2}{\rm{O}}}$) is considered for the oxides with the highest valence states at 700 °C under 1 atm. Thermodynamic data for constructing this diagram have been collected from the literature^2,41^ and the SGTE database^42^. **c**, Kinetic conception outlining the two main competing factors in achieving bulk alloys with defined microstructures from oxides, related in part to the present demonstrator Fe–Ni alloy class. The physical rationale of such a proposition lies in the difference between the oxide reduction temperature and the temperature at which complete densification is achieved (typically when *T*/*T*_m_ ≈ 0.75, where *T*_m_ is the bulk melting point^35^; see also Extended Data Fig. 1) in the corresponding metallic phase. The critical heating rate, *β*_c_, indicates the scenario in which complete oxides-to-alloy conversion and complete densification are simultaneously achieved. *β*_1_ and *β*_2_ sketch two heating rates slower than *β*_c_ as a guide to the eye.

Using H_2_-based redox reactions, our ‘one step oxides to bulk alloy’ operation (Fig. 1a) is aimed to reform the millennia-old multi-step alloy-making process (Fig. 1a, top) in three aspects: first, eliminating CO_2_ emission during fossil reductant-based metal extraction; second, reducing the energy cost of liquid processing^15,16^ that scales with melting temperatures; and third, exploiting the diffusion processes involved directly for compaction. The a priori feasibility of our sustainable alloy synthesis route is governed by the thermodynamic nature of the traditionally separated process steps that we merge here: metal extraction from oxides, atomic-scale mixing amongst the alloying elements and bulk material compaction by diffusion. (Fig. 1a, bottom). Our approach is based on a general thermodynamic design treasure map (Fig. 1b), using the two most important physical parameters involved: solid-state reducibility of oxides in H_2_, as quantified by $\Delta {G}_{{\rm{oxide}}}-\Delta {G}_{{{\rm{H}}}_{2}{\rm{O}}}$; and alloying capability, as quantified by the mixing enthalpy between substances (we exemplify Fe–X binary systems in Fig. 1b). Elements in the first and the fourth quadrants (Fe, Ni, Co and Cu) are those that can be fully reduced from their oxides at the solid state by H_2_, and the closer they locate to the ideal mixing line signifies the more preferential substitutional alloying capability with Fe (Fig. 1b). The thermodynamic validity of our design treasure map well aligns with both historical attempts on alloyed powders^17^ or nano-composite^18^ fabrication and the more recent literature on H_2_-based direct oxide reduction^19–21^.

For synthesizing not just powders or nanoparticles, but bulk alloys ready for applications, a secondary consideration promptly emerges: sufficient densification and reproducible bulk properties must be attained, which is governed by the kinetics of the underlying mass transport and microstructure formation mechanisms. This design rationale is essential as conventional multi-step alloy-making always requires a third step to reheat the as-cast materials for thermomechanical processing, endowing them with the desired microstructure–property combinations (Fig. 1a, top). Although a quantitatively precise kinetic design guideline relies on the targeted alloy system and product properties, a general conception could still be made, considering the overall interplay between oxides-to-alloy conversion and densification (Fig. 1c). In a theoretical framework encompassing temperature, time and conversion rate, these two phenomena divide the kinetic processing space into four regions, in which a critical heating rate is also involved: our ‘one step oxides to bulk alloy’ operation may be possible only in regions (i) and (ii) in which oxides-to-alloy conversion finishes before complete densification, and further temperature increase in region (i) leads only to salient microstructural coarsening. Regions (iii) and (iv) conversely suggest an incomplete oxides-to-alloy conversion, with moderate or noticeable densification depending on the temperature, respectively. Bearing these semi-quantitative thermodynamic–kinetic guidelines in mind, we next practise our ‘one step oxides to bulk alloy’ concept, aiming to synthesize bulk green Fe–Ni invar alloys as a demonstration. This endeavour is motivated by the substantial environmental costs^2,14^ associated with fabricating this class of attractive ferrous materials using the conventional extraction–alloying–thermomechanical processing three-step alloy-making procedure.

**Fig. 2:**
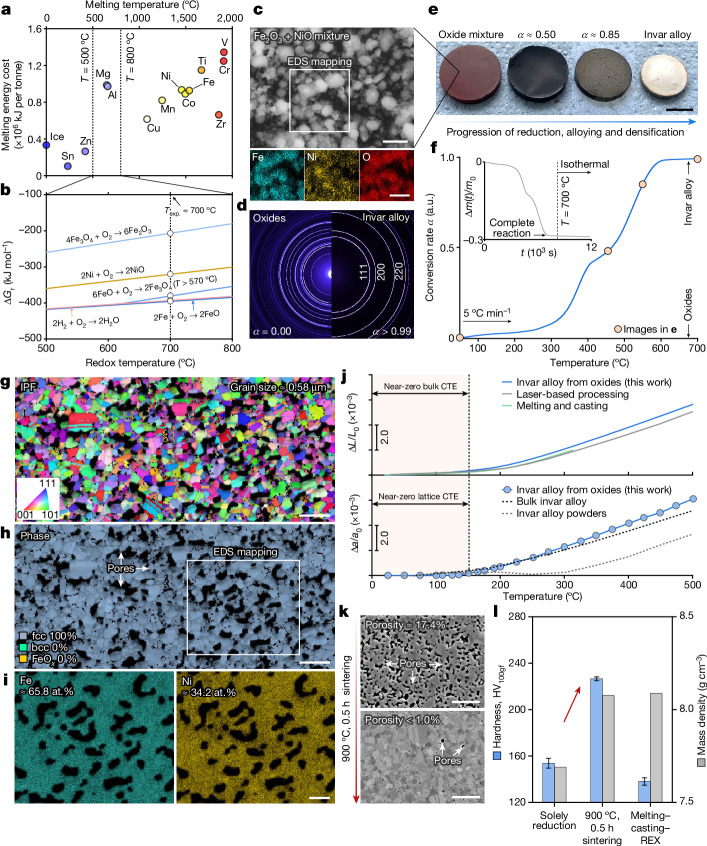
Synthesis kinetics, microstructure and thermal expansion property of the invar alloy fabricated from oxides. **a**, Minimum melting energy cost as a function of the melting point for common species. The estimation is conducted by adding the enthalpy change of heating up a certain substance from ambient temperature to its melting point and the enthalpy of fusion, that is, ${\Delta E}_{\min }={\int }_{{25}^{^\circ }{\rm{C}}}^{{T}_{{\rm{m}}}}{c}_{{\rm{p}}}\,{\rm{d}}T+{\Delta H}_{{\rm{f}}}$. Only the enthalpy of fusion is considered for ice. Thermophysical parameters for these estimations are acquired from the literature^41^. **b**, Predicted Ellingham–Richardson diagram for the oxides of Fe (Fe_2_O_3_, Fe_3_O_4_ and FeO) and Ni (NiO) under 1 atm (SGTE database^42^ and refs. ^2,41^). **c**, Secondary electron micrograph of the Fe_2_O_3_ + NiO powder mixture and the corresponding EDS maps. **d**, Two-dimensional SXRD diffractograms of the as-compacted oxide pellet (left) and the synthesized invar alloy (right). **e**, Macroscopic morphological evolution at different conversion rates. **f**, TGA curve showing the reduction kinetics. Inset: the instantaneous mass loss as a function of time. **g**, IPF map of the as-synthesized alloy (Σ3 annealing twin boundaries are excluded). **h**, The corresponding phase map. **i**, EDS mapping of Fe and Ni. **j**, Bulk thermal expansion (top, measured using dilatometer) and lattice thermal expansion (bottom, measured using in situ SXRD) results. The bulk and the lattice thermal expansion data for the invar alloys fabricated using different methods are reproduced from the literature^25,26,29,30^. **k**, Examples of microstructure tunability. Details of the microstructure after pressure-free sintering are provided in Extended Data Fig. 2. **i**, Vickers hardness and bulk mass density comparison among the green invar alloys synthesized from oxides and the one fabricated using the conventional melting–casting–recrystallization (REX) method. The recrystallization conditions for the invar alloy processed through melting–casting were also chosen as 900 °C, 0.5 h (around 70% cold-rolling thickness reduction), whose grain size is about 50 μm. a.u., arbitrary units; CTE, coefficient of thermal expansion. Scale bars, 1 μm (**c**, top and bottom, **i**); 5 mm (**e**); 2 μm (**g**,**h**); 5 μm (**k**).

**Extended Data Fig. 1:**
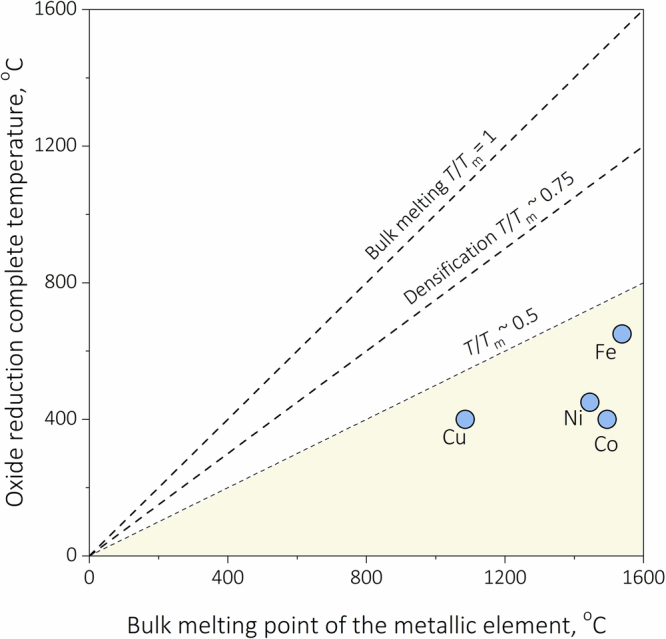
Complementary plot supporting the kinetic conception. Following the thermodynamic guideline discussed in main text Fig. 1b, the oxide reduction complete temperatures^51–53^ for Fe, Cu, Ni, and Co are compared with the bulk melting points. It is evident that for all these four elements, the corresponding oxides reduction by H_2_ completes below $0.75\,{T}_{{\rm{m}}}$, *i.e*. the homologous temperature at which significant densification takes place^35^.

Following the thermodynamic design treasure map (Fig. 1b), we first assess all the possible redox reactions in more quantitative depth. As seen in the calculated Ellingham–Richardson diagram, oxides of Fe and Ni with different valence states all locate above the 2H_2_ + O_2_ → 2H_2_O reaction beyond about 600 °C, suggesting the reducibility of these oxides by H_2_, and thereby, the formation of metallic Fe and Ni far below their melting points (Fig. 2a,b and Extended Data Fig. 1). To achieve an invar alloy, sufficiently homogeneous mixing between Fe and Ni is indispensable, and the extensive single-phase face-centred cubic (fcc) phase field in the Fe–Ni binary system alleviates such a concern: infinite solubility is present for alloys with more than 20 atomic percent (at.%) Ni above 600 °C. We next mixed Fe_2_O_3_ and NiO powders using low-energy ball milling (Fig. 2c,d, left), targeting the Fe–Ni ratio in the invar alloy and compacted them into pellets (Fig. 2e). With this precursor material, we mimic a naturally blended ore with all gangue oxides removed, motivating a proof-of-concept exploration. Secondary electron imaging and energy dispersive X-ray spectroscopy (EDS) confirm the homogeneous mixing of the two oxide powders (Fig. 2c) without discernible mechanical alloying.

Following the kinetic conception (Fig. 1c), we adopted a moderate heating rate of 5 °C min^−1^. When heated up in a H_2_ atmosphere, the pellet undergoes noticeable mass loss accompanied by volumetric shrinkage and colour change (Fig. 2e,f), underpinning the activation of redox reactions. Three stages can be discerned in the thermogravimetric analysis (TGA) (Fig. 2f): (1) reduction initiates at around 290 °C, with a linear increase in conversion rate until an inflection point at about 400 °C, where the reaction is momentarily impeded; (2) the conversion rate resumes to show a linear dependence on temperature in the 450–580 °C range; and (3) an asymptotic trend is present as the conversion rate approaches 0.95 until complete reduction. The presence of distinctive stages in the TGA curve implies the involvement of several redox and alloying micro-events^22,23^, as rationalized later. At complete reduction, the pellet exhibits a silvery surface, in distinctive contrast with the red-hematite appearance of its as-compacted counterpart (Fig. 2e). Synchrotron X-ray diffraction (SXRD) measurement further validates the single fcc phase constitution of the fully reduced pellet (Fig. 1d, right), in which no remaining oxide phase is detected. The lattice constant of this fcc phase is 3.60 Å. Even considering the uncertainties involved in various measurement techniques, such a lattice constant value agrees well with the literature data for Fe–Ni invar alloys^24–26^ (about 3.60 Å); however, it is notably larger than that of pure fcc-Ni^24,27,28^ (about 3.52 Å). This distinction evidences substitutional alloying between Fe and Ni during the solid-state redox synthesis, staying consistent with our theoretical anticipation. With this, we prove that the traditionally separate steps of metal extraction and mixing can be merged in a single operation under suitable thermodynamic–kinetic boundary conditions (Fig. 1a).

To assess the ‘oxides to bulk invar alloy’ proposition, we characterize the microstructure and examine the thermal expansion property of the synthesized alloy. Figure 2g shows the electron backscatter diffraction (EBSD) inverse pole figure of the as-synthesized alloy, revealing an equiaxed grain morphology with a fine average grain size of approximately 0.58 μm. Despite the sub-micro-scale porosity owing to the redox-catalysed mass loss and incomplete densification, the microstructure exhibits a single fcc phase constitution (Fig. 2h) without detectable body-centred cubic (bcc) or residual oxide phase at the spatial resolution limit of EBSD (around 50 nm). EDS maps also verify the grain-level uniform distribution of Fe and Ni (Fig. 2i), the contents of which closely follow the conceived values of an invar alloy (Fe–34.8 at.% Ni). The thermal expansion property of the synthesized alloy is next examined using both dilatometry and in situ SXRD. A discernible near-zero coefficient of thermal expansion region is present in the 25–150 °C range for both the bulk and the lattice thermal expansion responses (Fig. 2j). This invar property aligns well with the literature data^25,26,29,30^ for invar alloys fabricated through the conventional melting–casting–thermomechanical processing routes and even the state-of-the-art laser-based processing methods, again validating our one-step sustainable bulk alloy synthesis approach (Fig. 1a).

**Extended Data Fig. 2:**
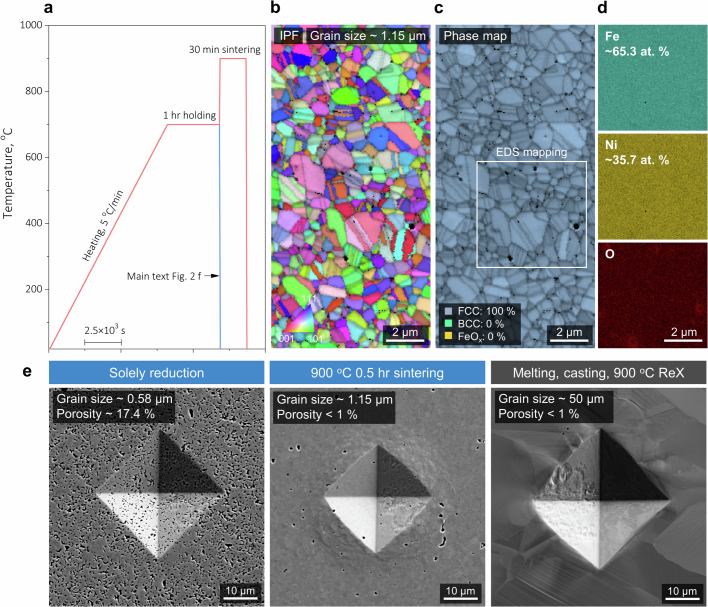
Microstructure of the invar alloy fabricated with an additional pressure-free sintering step and bulk property assessment. **a**, Comparison between the two temperature profiles. The one utilized in main text Fig. 2f is sketched in blue, and the additional pressure-free sintering step is added right after the isothermal holding period at 700 °C. To hinder grain growth, a fast heating rate of 300 °C/min is applied to achieve 900 °C. **b**, IPF map showing the grain morphology after pressure-free sintering and the average grain size (~1.15 μm) is obtained by excluding the Σ3 annealing twin boundaries. **c**, Phase map validating the single-phase FCC microstructure where no detectable BCC or residual oxide phase is present. **a** and **b** are both overlapped with image quality maps and datum points with confidence index small than 0.1 have been removed. **d**, EDS maps taken across multiple grains validating the uniform distribution of Fe and Ni, and their contents are consistent with the designed values. **e**, Vickers hardness measurements of the bulk green invar alloys synthesized directly in one step from oxides (left and middle micrographs) and the one fabricated using the convention melting-casting-recrystallization method (right micrograph). The highest Vickers hardness of 226.6 ± 1.6 HV_100gf_ is seen in the green invar alloy synthesized with an additional pressure-free sintering step, which is ~1.5 times the hardness of the coarse-grain invar alloy fabricated using the conventional melting-casting-recrystallization route (138.0 ± 3.2 HV_100gf_). Despite the presence of excessive porosity in the as-reduced green invar alloy (~17.4 %), no cracking event is observed at the corners of the indent, and its hardness reaches 153.7 ± 4.2 HV_100gf_, revealing its load-bearing capacity, which may even serve to inspire meso-porous bulk invar foam design as a future work avenue.

The microstructure reported here is mainly aimed to showcase the validity of the ‘one step oxides to bulk alloy’ synthesis concept (Fig. 1), yet, much more diverse microstructure–property combinations can be realized by different kinds of integrated reduction, compaction and microstructure design treatments, implied by our kinetic conception (Fig. 1c). As a simple example, we present in Fig. 2k the possibility of achieving a fine-grain fully densified invar alloy by adding a 0.5 h pressure-free sintering step at 900 °C (correspond to region (i) in Fig. 1c). The average porosity drops from about 17.4% to less than 1%, whereas the average grain size is maintained as about 1.15 μm (Extended Data Fig. 2) and almost all the intercrystalline pores are annihilated, resulting in a bulk mass density nearly identical to that of the invar alloy fabricated through the conventional melting–casting–recrystallization route (Fig. 2l). Because of the salient grain size refinement benefited from the oxide reduction–compaction operation at moderate temperatures, our fully densified invar alloy exhibits a Vickers hardness under 100 g force of 226.6 ± 1.6 HV_100gf_ (Fig. 2l), far exceeding that of the coarse-grained invar alloy obtained from the conventional processing route (138.0 ± 3.2 HV_100gf_). The foregoing analyses unambiguously substantiate the proposed sustainable alloy synthesis concept (Fig. 1a, bottom) that near-optimized microstructure–bulk property combinations are attained from oxides fully at the solid state. The successful synthesis of the bulk Fe–Ni alloy further reflects the essence of three core physical phenomena at play, as implied by the thermodynamic guideline and the kinetic conception: carbon-free oxide reduction, substitutional alloying and densification for microstructure–property design, altogether motivating mechanistic explorations, as shown next, starting from the phase constitution evolution during reduction.

**Fig. 3:**
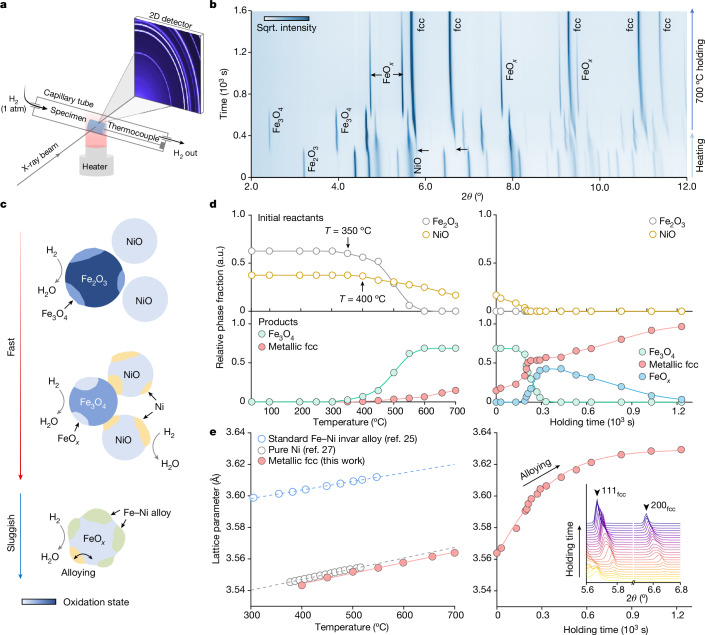
In situ SXRD assessment of the synthesis mechanisms. **a**, Schematic of the experimental set-up and the sample condition. The actual configuration of the experimental instruments is provided in Extended Data Fig. 5. **b**, Two-dimensional (2D) phase evolution map plotted as a function of time showing the oxide reduction pathway. **c**, Schematic of the multi-step reduction mechanisms. The colour scale applied in all the oxide phases quantitatively indicates the oxidation state (that is, relative oxygen content). **d**, Relative phase fraction evolution determined through Rietveld refinement. Note that because of the mass loss during the redox reaction (that is, phase fraction of H_2_O is unmeasurable by SXRD), datum points in the left and right panels cannot be used to back-derive absolute phase fraction with respect to the reactants. In addition, because of the substantial reaction boundary condition differences between in situ SXRD and TGA, directly correlating these microscopic phase fraction evolution processes with the global conversion rate measurements may not be possible. **e**, Lattice constant change of the metallic fcc phase observed in the present experiment. Literature data for the pure Ni (ref. ^27^) and the standard Fe–Ni invar alloy^25^ are also included in the left panel as references. Inset: the peak shift of 111 and 200 peaks in the metallic fcc phase during 700 °C isothermal holding.

**Extended Data Fig. 3:**
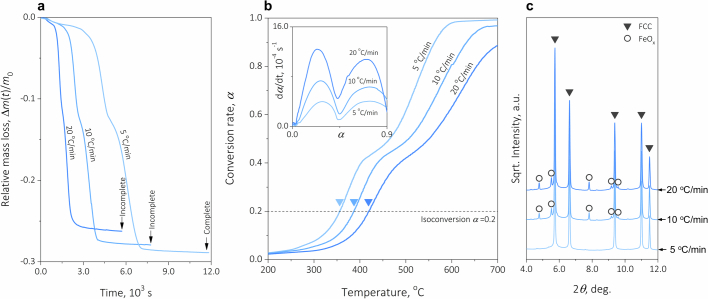
Conversion rates measured using different heating rates. **a**, Relative mass loss over time measured using 5, 10, and 20 °C/min heating rates. To stay consistent with the main text results (Fig. 1g), all the specimens were heated up to 700 °C, followed by 1 hr isothermal holding period. **b**, Conversion rate curves as a function of temperature acquired using three different heating rates. The same conversion rate is reached at higher temperature as the heating rate increases (see for example, the $\alpha =0.2$ isoconversion line as a guide to the eye), signifying the thermal activation nature of the operating reduction mechanisms. Inset of **b** reveals the time derivative of the conversion rate with respect to the progression of the reduction. Two peaks are present in all three ${\rm{d}}\alpha /{\rm{d}}t$ curves, proving the involvement of multiple reduction micro-events. These results are further utilized to determine effective activation energy following isoconversional principle^31,32^. **c**, SXRD diffraction patterns of the pellets after the measurements. It is seen that complete reduction is achieved only with 5 °C/min heating rate, while discernible amounts of FeO_x_ oxide remain in the pellets when higher heating rates were utilized. The presence of the residual FeO_x_ phase aligns well with the in situ SXRD results revealed in main text Fig. 3, where the reduction of FeO_x_ is recognized as the most sluggish reaction mechanism.

**Extended Data Fig. 4:**
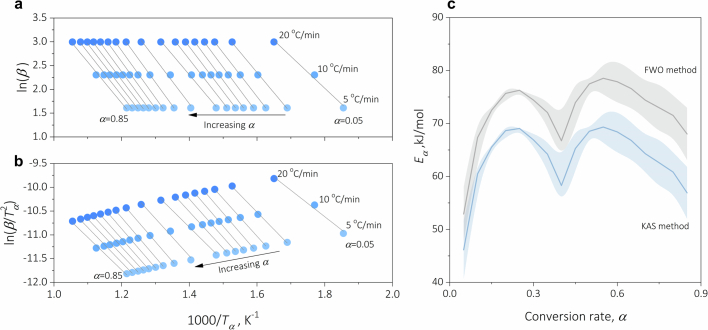
Determination of the effective activation energy using isoconversional principle. **a**, Flynn−Wall−Ozawa (FWO) method^54,55^. **b**, Kissinger-Akahira-Sunose (KAS) method^56,57^. **c**, Calculated effective activation energy (${E}_{\alpha }$) as a function of conversion rate ($\alpha$). The theoretical basis of these analyses roots in the isoconversional principle^31,32^, presuming that for any given chemical reaction, the reaction rate at a constant extent of conversion should only depend on temperature. It is hence possible to evaluate the effective activation energy (${E}_{\alpha }$) for individual conversion rate ($\alpha$) in a model-free manner, without priori assumption of the reaction model. For a non-isothermal process (in this case, continuous heating), the generalized kinetic rate theory states: ${\rm{d}}\alpha \,/{\rm{d}}t=k(T)f(\alpha )$, where $k(T)$ is the rate constant, whose temperature dependency closely follows Arrhenius law: $k(T)={k}_{0}\exp (\,-\,Q/{RT})$, and $f(\alpha )$ represents a complex function of $\alpha$. When a constant heating rate is utilized during a TGA measurement: $\beta ={\rm{d}}T\,/{\rm{d}}t$, a further correlation can be set: ${\rm{d}}\alpha \,/{\rm{d}}T=({\rm{d}}\alpha \,/{\rm{d}}t)({\rm{d}}t\,/{\rm{d}}T)=(1/\beta )({\rm{d}}\alpha \,/{\rm{d}}t)$ and the generalized rate equation hence becomes: ${\rm{d}}\alpha /{\rm{d}}T=({k}_{0}/\beta )\exp (\,-\,Q/{k}_{{\rm{B}}}T)f(\alpha )$, whose temperature integration is: $g(\alpha )=\int (1/f(\alpha )){\rm{d}}\alpha =({k}_{0}/\beta )\int \exp (\,-\,Q/{k}_{{\rm{B}}}T){\rm{d}}T$. The FWO method takes the integration with Doyle’s approximation^54,55^, leading to: $\mathrm{ln}(\beta )=\mathrm{ln}({k}_{0}\,{E}_{\alpha }/Rg(\alpha ))-5.3305-1.052({E}_{\alpha }/R{T}_{\alpha })$, thus by evaluating the slope on the $\mathrm{ln}\left(\beta \right)v.s.\left(1/{T}_{\alpha }\right)$ plot per each $\alpha$, the effective activation energy can be obtained. The KAS method^56,57^, on the other hand, takes a linear integration that brings about: $\mathrm{ln}(\beta /{T}_{\alpha }^{2})=\mathrm{ln}({k}_{0}\,{E}_{\alpha }/Rg(\alpha ))-({E}_{\alpha }/R{T}_{\alpha })$, and thus on the $\mathrm{ln}(\beta /{T}_{\alpha }^{2}){v}.s.\,(1/{T}_{\alpha })$ the effective activation energy can be determined at each $\alpha$. The presence of a non-constant ${E}_{\alpha }$ throughout an $\alpha$ range of 0.05–0.85 proves the onsets of more than one reaction process at play, as further revealed in the main text using in situ SXRD.

Resorting to the conversion rate curve measured by TGA (Fig. 2f) and the predicted Ellingham–Richardson diagram (Fig. 2b), reduction of the Fe_2_O_3_ + NiO mixed oxide should involve several steps, each dictated by the thermodynamics of the redox reaction of the individual oxide. Model-free assessments using the iso-conversional principle^31,32^ also validate the pronounced dependence of the effective activation energy (*E*_*α*_) on the local conversion rate (*α*), indicating the presence of multiple reaction micro-events (Extended Data Figs. 3 and 4). To consolidate this mechanistic proposition, we next performed in situ SXRD measurements (Fig. 3a), characterizing the phase constitution change over time (Supplementary Video 1). The progression of the reaction is shown in Fig. 3b and the schematics in Fig. 3c. The stepwise nature of the reduction process is evident (Fig. 3b), as seen from the quantified relative phase fraction change (Fig. 3d). The first reduction step is Fe_2_O_3_ → Fe_3_O_4_, which initiates at around 350 °C, leading to the continuous increase of the Fe_3_O_4_ phase fraction to about 0.68 at 600 °C (Fig. 3c, top). The reduction of NiO occurs at a slightly higher temperature of around 400 °C, whose fraction monotonically decreases as the reaction advances. The metallic fcc phase emerges from 400 °C (later proved as pure Ni) onwards and its fraction continuously increases to approximately 0.15 as the heating process terminates at 700 °C. On isothermal holding (Fig. 3c, middle, and 3d, right), the NiO phase completely diminishes, associated with the increase of the metallic fcc phase fraction. The Fe_3_O_4_ phase fraction remains momentarily constant, followed by the onset of the Fe_3_O_4_ → FeO_*x*_ reaction, leading to a peak FeO_*x*_ phase fraction of about 0.43. For the rest of the isothermal holding period, the metallic fcc phase fraction keeps increasing and vice versa for the FeO_*x*_ phase fraction (Fig. 3c, bottom), which accounts for the most sluggish step.

We next focus on solid-state substitutional alloying, the central mechanism in synthesizing the invar alloy. Our rationalization is grounded in the compositionally dependent lattice constants of fcc-structured Fe–Ni alloys. Comparing the lattice constant of the metallic FCC phase obtained here and those of pure Ni (ref. ^27^) and a standard invar alloy^25^ (Fig. 3e, left), it is evidenced that pure Ni is the initial metallic phase reduced from the oxide mixture (Fig. 3c, middle). The lattice constant of the metallic fcc phase increases linearly in the 400–700 °C temperature range, consistently aligning with the literature report^27^ for pure Ni. During isothermal holding (Fig. 3e, right), however, a power-function-like increase in the lattice constant of this phase is present, which eventually plateaus at the lattice constant value of the standard invar alloy. This trend unequivocally reflects the progression of substitutional alloying, which necessitates Fe dissolution into the pure Ni-phase formed earlier and coincides with the prolonged reduction of the FeO_*x*_ phase.

**Fig. 4:**
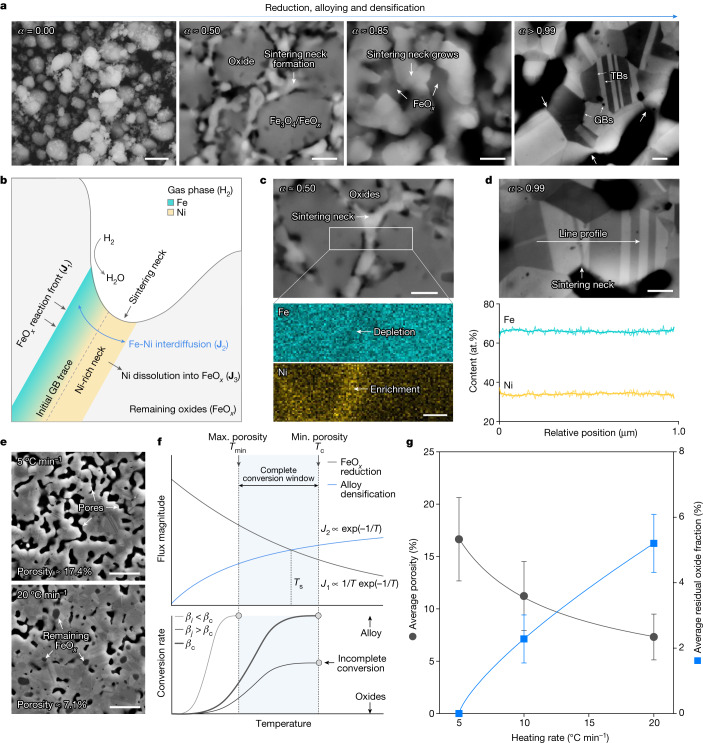
Microstructural analyses at different conversion rates and kinetic mechanism explorations. **a**, Observations of sintering neck development at different synthesis stages (corresponds to Fig. 2f). **b**, Schematic of the critical mass transport process. **c**, EDS analyses of Fe and Ni distribution across a neck at a global conversion rate of around 0.5. **d**, EDS line profile of Fe and Ni distribution across a neck at a complete reduction. **e**, Representative secondary electron micrographs of two specimens synthesized using slow and fast heating rates. **f**, Schematic of the temperature dependency of the two main competing fluxes. Here, *J* and *β* denote the flux magnitude of individual mass transport mechanism and the heating rate, respectively. Assuming a constant concentration gradient, the magnitude of the interdiffusion flux facilitating densification, scales with temperature following the Arrhenius law^43,44^, that is, *J*_2_ ∝ exp(−1/*T*). The flux magnitude for FeO_*x*_ reduction, takes the form *J*_1_ ∝ exp(−*E*_*a*_/*T*)[1 − exp(−Δ*G*_r_/*T*)]), as suggested by transition state theory^44–46^, where *E*_*a*_ and Δ*G*_r_ are the activation energy and the thermodynamic driving force, respectively. The reduction of FeO_*x*_ in H_2_ gas exhibits the smallest thermodynamic driving force (Δ*G*_r_ ≈ 0, with notable backward reaction^38,39,47^; see also the Ellingham–Richardson diagram in Fig. 2b), allowing us to linearize its temperature dependency to *J*_1_ ∝ 1/*T* exp(−1/*T*). **g**, Trade-off between porosity and residual oxide content observed in the specimens obtained using different heating rates. Here the error bars represent the standard deviations. Scale bars, 1 μm (**a**, for *α* ≈ 0.00); 200 nm (**a**, for *α* ≈ 0.50, *α* ≈ 0.85 and *α* > 0.99); 150 nm (**c**); 200 nm (**d**); 2 μm (**e**).

Blending the foregoing thermodynamically oriented insights, we finally explore the governing kinetic phenomena that contribute to the successful invar alloy synthesis and microstructure design directly from the oxides. We suggest that the most prominent rate-limiting process lies in the interplay between interdiffusion-facilitated densification and the FeO_*x*_ reduction. Pronounced densification occurs during the synthesis, as seen from the considerable volumetric shrinkage of the pellet (Fig. 2e), which follows our kinetic conception (Fig. 1c). As oxide reduction inherently involves volumetric shrinkage^33^, we present in Extended Data Fig. 6 theoretical calculations, showing that more than 30% of the total volumetric shrinkage is ascribed to sintering-driven densification. Zooming in from macro to micro, we show in Fig. 4a, that the development of the sintering necks corresponds to the material state in Fig. 2e. At a global conversion rate of about 0.5, the formation of metallic interparticle necks initiates and they notably grow as the redox reaction advances to a global conversion rate of about 0.85. Evident densification concurrently operates and the initial necks merge, bringing about multiple grain and annealing twin boundaries at the complete reduction stage. According to the multi-step reaction mechanisms shown earlier, when all the Fe_2_O_3_ is reduced to FeO_*x*_ and NiO to pure Ni, the global conversion rate is about 0.52, implying a direct kinetic overlap among further FeO_*x*_ reduction, densification and microstructure design.

Following these experimental observations, and the earlier overall kinetic conception presented in Fig. 1c, a mechanistic sketch is proposed to detail the governing kinetic processes (Fig. 4b). Two competing mechanisms are considered, the reduction of FeO_*x*_ by H_2_ (flux **J**_1_) and densification facilitated by Fe–Ni interdiffusion (flux **J**_2_). Here we conceive an Ni-rich metallic interparticle neck at the initial state, as supported by the leading NiO → Ni reaction step evidenced by in situ SXRD (Fig. 3) and EDS measurements in Fig. 4c. The role of interdiffusion is remarkable because the synthesized invar alloy shows a grain-level uniform distribution of Fe and Ni (Fig. 2i), and Fig. 4d also underpins the chemical homogeneity across a typical sintering neck. Although Ni may also re-dissolve into the FeO_*x*_ phase^34^ (flux **J**_3_), atom probe tomography results (Extended Data Fig. 7) confirm the negligible role of this process. Under the microstructural state shown in Fig. 4b, when the FeO_*x*_ reduction initiates, the freshly formed Fe tends to dissolve into the Ni-rich neck and the eminent local concentration gradient further drives interdiffusion, enabling substitutional alloying (Fig. 3e, right). We note that this process may be accomplished through a transient Fe-rich reaction front (several nm thick) at the FeO_*x*_/Ni-rich neck interface, without bulk Fe nucleation. This might be responsible for the absence of any metallic Fe phase (bcc) throughout the SXRD diffractograms (Fig. 3b) and detailed atomic-level characterization is advised as future work.

The densification contribution through interdiffusion is also prominent because it naturally facilitates mass transport to the sintering neck^35–37^ and is confirmed to be more effective than Ni self-diffusion (Extended Data Fig. 6). Upon densification, open pores annihilate, resisting the reduction of FeO_*x*_ because of the retardation of effective H_2_ transport and the release of H_2_O (refs. ^38,39^). This thermodynamic–kinetic jointly governed process implies the crucial role of heating rate in the synthesis: as compared in Fig. 4e, the specimen obtained using a faster heating rate (20 °C min^−1^) contains considerable residual FeO_*x*_ phase with reduced porosity, whereas successful synthesis of the invar alloy is achieved with a slower heating rate (5 °C min^−1^; Extended Data Figs. 3 and 8). This distinction can be explained by the temperature dependency of the fluxes for interdiffusion (**J**_2_) and FeO_*x*_ reduction (**J**_1_), as shown in Fig. 4f, in which a crossover point is expected at temperature *T*_s_ (see legend of Fig. 4 for semi-quantitative rationalization). Below this temperature, interdiffusion-driven densification is modest compared with the eminent FeO_*x*_ reduction. With a sufficiently slow heating rate (*β*_*i*_), complete conversion can be attained (Fig. 4f, bottom), leaving excessive porosity in the microstructure (see also Fig. 1c). Conversely, densification becomes more predominant above *T*_s_, resisting the inherently sluggish FeO_*x*_ reduction, particularly when conversion remains incomplete before *T*_s_ under a fast heating rate (*β*_*j*_). These theoretical analyses are supported by experimental observations, in which an evident trade-off is observed between porosity and residual oxide content with respect to heating rate (Fig. 4g). With the foregoing considerations, we also anticipate a complete conversion window bounded by the minimum temperature to activate FeO_*x*_ reduction and the complete conversion temperature corresponds to the fastest possible heating rate (*β*_c_). Within such a temperature window, a tunable design of porosity and grain size is possible (see also Fig. 1c), diversifying the microstructure design space for the ‘one step oxides to alloy’ synthesis approach (Fig. 1a).

The thermodynamic–kinetic insights mentioned above not only theorize the successful synthesis of bulk Fe–Ni invar alloys from oxides, used here as a demonstrator example, but open a new general paradigm of fabricating metallic alloys directly from oxides through a one-step solid-state process (Fig. 1). To demonstrate the universality of this synthesis route, we exemplify in Extended Data Fig. 9 a one-step synthesis also of a ternary fine-grain bulk Fe_63_Ni_32_Co_5_ super invar alloy^40^ directly from oxides. To generalize our synthesis method to industrial practice, three core factors require consideration. First, effective removal of gangue oxides from natural ores through mechanical separation and hydrometallurgical purification. Second, balancing H_2_ partial pressure and process temperature. To alleviate the high H_2_ partial pressure (>0.75) required for complete reduction at 700 °C, improving gas convection in the countercurrent flow furnace or slightly increasing the process temperature up to 800–900 °C is recommended for large-scale production. Third, complete pore annihilation. Although a 0.5-h pressure-free sintering step can reduce the porosity level below 1% for laboratory-scale specimens (Fig. 2k), industrial-scale production might necessitate overlaid hot isostatic pressing to improve the structural integrity of the final product. Finally, for a rough estimation (Extended Data Fig. 10), our ‘one step oxides to bulk alloy’ operation may reduce around 41% energy cost (about 6.97 GJ tonne^−1^) compared with the traditional multi-step alloy-making approach.

In summary, we report here a redox-inspired sustainable alloy design concept fulfilling one-step synthesis of bulk alloys directly from oxides. Following the thermodynamic guideline and the integrated kinetic conception, we applied this approach to the fabrication of bulk Fe–Ni invar alloys with microstructure–bulk property combinations that are ready to be deployed in real-world applications. The as-synthesized alloy not only exhibits a near-zero thermal expansion property aligning well with the invar alloys fabricated using the traditional multi-step metal extraction, liquid alloying and thermomechanical processing routes but is also accessible to wide microstructure tunability. The universality of our approach, however, goes beyond the specific scope of Fe–Ni binary invar alloy synthesis: the same concept can be extended (1) to various dilute oxide-bonded transition metals and (2) to even highly contaminated oxidized feedstocks of diverse origins. This approach also dissolves some of the classical boundaries between extractive and physical metallurgy, inspiring direct conversion from oxides to application-worthy products in one single solid-state operation.

## Methods

### Oxide pellet fabrication and multi-scale microstructural characterization

Standard 325 mesh Fe_2_O_3_ and NiO powders (purity >99.7%) were used as the raw materials for the synthesis. The powders were weighed aiming for the Fe–Ni ratio of the standard Fe–36 wt % Ni (equivalent to Fe–34.8 at.% Ni) invar alloy and subjected to low-energy ball milling (250 rpm for 5 h, ball-to-powder ratio 5:1) to achieve homogenous mixing. No detectable mechanical alloying took place during the mixing process, as evidenced by synchrotron X-ray measurement. The as-milled powders were compacted into individual cylindrical green bodies with a diameter of about 13 mm and a thickness of around 2 mm using a pellet press die with about 3.75-tonne hydraulic force. The mass of each pellet was consistently kept to about 1.0 g intended for thermogravimetry measurements. No additional cold isostatic pressing was involved in the present study.

Meso-scale microstructural characterizations, including secondary electron imaging, BSE imaging and EBSD measurements were all conducted in a Zeiss Merlin scanning electron microscope. The raw EBSD diffractograms were collected using an acceleration voltage of 15 keV and a beam current of 5.0 nA at a working distance of 18 mm. An Orientation Imaging Microscopy software was opted for quantitative analysis of the EBSD results. Specimens for the quantitative analysis were prepared following the standard metallographic routes: cross sections were cut from the pellet using a low-speed diamond wire saw, ground on a series of SiC abrasive papers and polished on diamond suspension with 3 μm and 1 μm particle size. Final polishing was accomplished using 40 nm colloidal silica with a few drops of soap for around 30 min to ensure high surface quality for the EBSD measurements. Phase constitution and crystal structure of the presenting phases were analysed using SXRD at beamline P02.1, PETRA III of DESY (X-ray beam wavelength of 0.20735 Å). Details of these ex situ measurements along with the data analysis method closely resemble the in situ experiments detailed in the later section. Bulk thermal expansion responses of the synthesized alloys were analysed using a dilatometer (TA Instruments, DIL805 AD). Rectangular beams with dimensions of 7.5 × 4.0 × 1.0 mm^3^ were prepared for these measurements using electrical discharge machining. Vickers hardness measurements were carried out on a NEMESIS 5100 hardness tester, through which at least nine indents were performed on each specimen using 100 gf with a dwell time of 15 s. The bulk mass density of the specimens was measured following Archimedes’ principle with an electronic balance of 0.01 mg precision.

To understand the extent of Ni re-dissolution into the FeO_*x*_ phase, atom probe tomography analyses were performed. The atom probe tomography samples were prepared from a pellet with more than 0.90 global conversion rate (20 °C min^−1^ heating rate), which exhibits discernible residual FeO_*x*_ oxide phase (Extended Data Fig. 3). An FEI Helios NanoLab600i dual-beam scanning electron microscope was adopted for lamellar lift-out and tip granular milling. A Cameca LEAP 5000XS instrument was used to collect the data in the laser-pulsing mode. The laser frequency and pulse energy were chosen as 40 pJ and 200 kHz and the working temperature was kept as 50 K during the acquisition. The 3D atom maps and data post-processing were carried out in an AP Suite 6.1 software.

### Thermogravimetry assessment of oxide reduction kinetics in H_2_ gas

The reduction kinetics was assessed using an in-house thermal balance equipped with a temperature-programmed infrared heating furnace, for which the detailed configuration is shown in an earlier study^48^. To ensure the precision of the measurement and to also alleviate fluctuations in the mass balance, the system was stabilized overnight in moderate H_2_ gas flow (<5 l h^−1^, purity 99.999%). During the test, the pre-compacted oxide pellet was heated up to 700 °C using different heating rates (5, 10 and 20 °C min^−1^) in 10 l h^−1^ H_2_ gas flow (purity 99.999%) and isothermally held for 1 h before cooling down to ambient temperature. The instantaneous mass of the pellet was continuously recorded during the test, based on which the conversion rate can be calculated as $\alpha \left(t\right)=\frac{m\left(t\right)-{m}_{0}}{{m}_{\infty }-{m}_{0}}$. Here, *m*(*t*) and *m*_0_ are the instantaneous and the initial masses. The theoretical mass at complete reduction, *m*_∞_, was estimated by assuming a complete reaction of Fe_2_O_3_ → Fe and NiO → Ni within the pellet. Specimens at intermediate stages of the reaction (Figs. 1f and 4a) were obtained by interrupting the reduction using high flow rate Ar gas (purity 99.999%) at conceived global conversion rates followed by fast cooling (in this case, shutting down the heating furnace). For specimens intended for densification study, an extra 30 min annealing was set at 900 °C right after the isothermal holding period at 700 °C, which closely mimics the condition of pressureless sintering. Representative sketches of the temperature profiles can be found in Extended Data Figs. 2 and 9.

### In situ SXRD study

**Extended Data Fig. 5:**
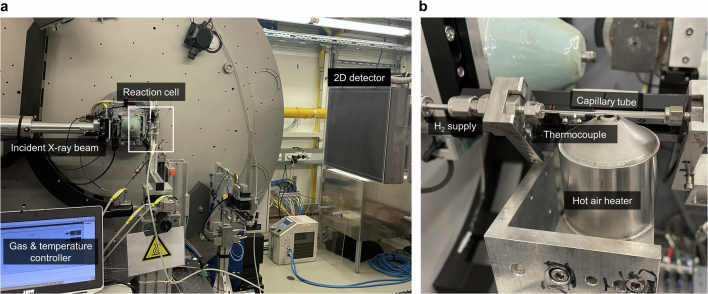
Experimental setup of the in situ synchrotron X-ray measurement. **a**, An overview of the experimental instrument. **b**, A closer view of the reaction capillary tube and the hot air heater. A standard type-K thermocouple is placed right next to the specimen in the capillary tube and the hot air heater is centered with respect to the position of the specimen.

**Extended Data Fig. 6:**
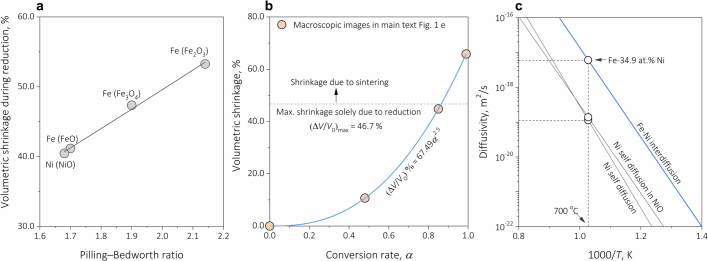
Theoretical calculations complementing the densification discussion in the main text. **a**, Pilling-Bedworth ratios^33,58^ (${R}_{{\rm{PB}}}$) for all the possible oxides involved, including Fe_2_O_3_, Fe_3_O_4_, FeO, and NiO. Such a quantity reflects the molar volume ratio between a pure metal and its oxide: ${R}_{{\rm{PB}}}={V}_{{\rm{oxide}}}/n{V}_{{\rm{metal}}}$, here $n$ accounts for the metal atom stoichiometry per molar volume of the oxide. For the reduction process considered here (turning oxide to metal), the relative volumetric shrinkage can be theoretically predicted as: $(\Delta V\,/{V}_{{\rm{oxide}}})=1-1/{R}_{{\rm{PB}}}$. By opting a simple composite theory, we further estimated that the maximum possible volumetric shrinkage when per unit mass of Fe_2_O_3_ + NiO mixtures is completely reduced to metallic state is 46.7 %. **b**, Comparison between the measured volumetric shrinkage of the pellet at different reduction stages and the theoretical maximum possible value. Excessive Volumetric shrinkage is clearly seen, which accounts for more than 1/3 of the total volumetric shrinkage, confirming the pronounced effect of sintering-driven densification during the reduction process. **c**, comparison between the Fe-Ni interdiffusion coefficient and the Ni self-diffusion coefficient. Here the interdiffusion coefficient is determined through Darken’s second equation^43^: $\widetilde{{\mathscr{D}}}={c}_{{\rm{Fe}}}\,{D}_{{\rm{Ni\; in\; Fe}}}+{c}_{{\rm{Ni}}}\,{D}_{{\rm{Fe\; in\; Ni}}}$, where ${D}_{{\rm{Ni\; in\; Fe}}}$ and ${D}_{{\rm{Fe\; in\; Ni}}}$ are the tracer diffusion coefficient of Ni in Fe and Fe in Ni. The cation self-diffusion coefficient in NiO is also included as a reference. Raw data for the calculations shown in **c** are obtained from the literature^41,59,60^.

**Extended Data Fig. 7:**
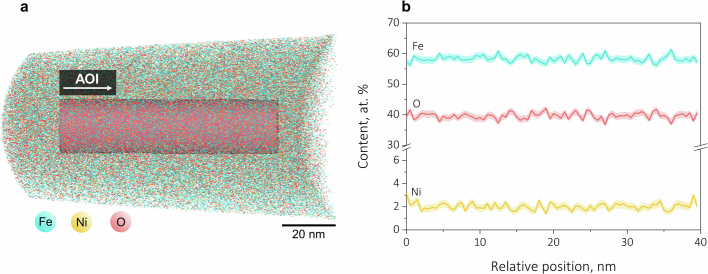
Atom probe tomography results assessing the extent of Ni redissolution into FeO_x_. **a**, The 3D reconstructed atom distribution map for Fe, Ni, and O. This APT sample was taken from a pellet synthesized using 20 °C/min heating rate where noticeable FeO_x_ is remained (see Extended Data Fig. 2c for the SXRD results). **b**, Fe, Ni, and O content acquired within a cylindrical area of interest (AOI) shown in **a**. The maximum amount of Ni in the FeO_x_ barely exceeds ~3 at. %, confirming the subtle role of Ni redissolution, which validates the kinetic proposition discussed in main text Fig. 4b. We note that the Fe-O stoichiometry ratio is not rigorously unity, which is mostly due to the oxygen loss during APT measurement. The O content measured for the FeO_x_ phase is consistent with similar analysis in the literature^61^.

**Extended Data Fig. 8:**
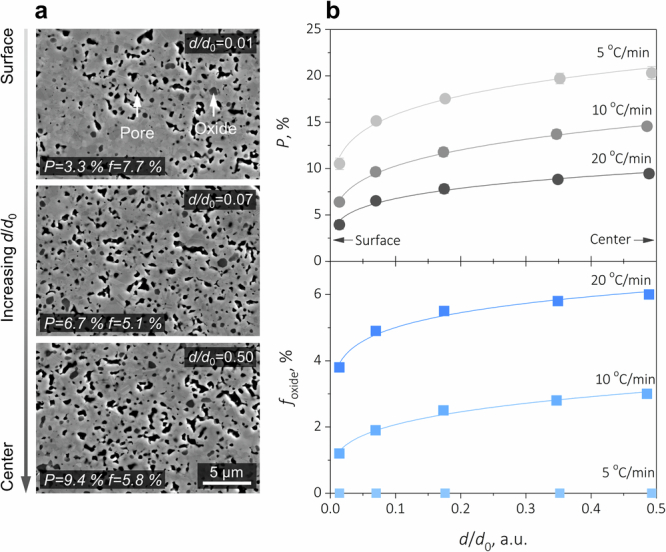
Quantitative microculture analyses assessing the role of heating rate. **a**, To account for potential inhomogeneity of porosity distribution, we examined the microstructure at different positions through thickness. Here the quantity $(d\,/{d}_{0})\in (0,\,0.5)$ is introduced to enable systematic comparisons amongst different samples: $d$ denotes the relative distance to the outer surface and ${d}_{0}$ the total thickness (close to 1.5 mm for all samples). SE images in **a** exemplify the microstructures of the specimen synthesized using 20 °C/min heating rate. **b**, Evolution trends of porosity and residual FeO_x_ oxides under three different heating rates corresponding to the results in Extended Data Fig. 3. Despite the subtle gradient in the thickness direction, the increase in heating rate systematically elevates the residual FeO_x_ content (incomplete reduction) and reduces the porosity (more eminent densification).

**Extended Data Fig. 9:**
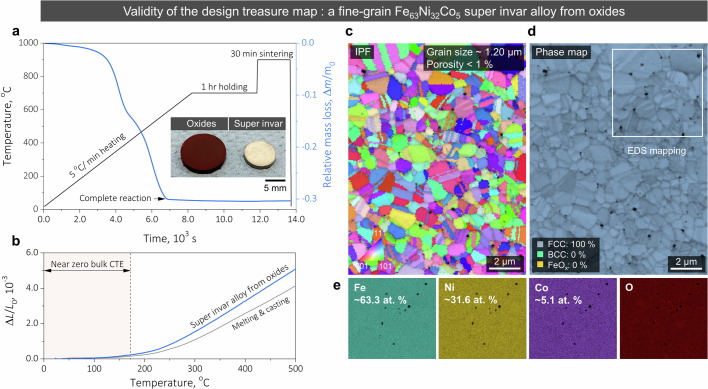
Validity of the proposed design treasure map. **a**, TGA analysis of the synthesis process. Following the exact synthesis route described in the main text, we start with green compacted pellet of Fe_2_O_3_, NiO, and Co_3_O_4_ powders aiming for the super invar alloy composition^40^, Fe_63_Ni_32_Co_5_ at. %. Following the kinetic guideline discussed in main text Fig. 4 we adopted a consistently slow heating rate of 5 °C/min, followed by 1 hr holding at 700 °C, and 30 min pressure free sintering at 900 °C. **b**, Bulk thermal expansion response of the synthesized alloy measured by dilatometer. A noticeable near zero coefficient of thermal expansion (CTE) region is present, which well aligns with the literature data^62^ of the same alloy fabricated through conventional melting and casting routes. **c**, The synthesis alloy exhibits a fine-grain fully densified microstructure with an average grain size of ~1.20 μm (excluding the Σ3 annealing twin boundaries). **d**, Phase map showing the single FCC-phase constitution of the alloy where BCC or residual FeO_x_ phase is not detected. **e**, EDS maps confirming the grain-level uniform distribution of the alloying elements Fe, Ni, and Co and their contents aligns with the conceived values for a Fe_63_Ni_32_Co_5_ super invar alloy.

**Extended Data Fig. 10:**
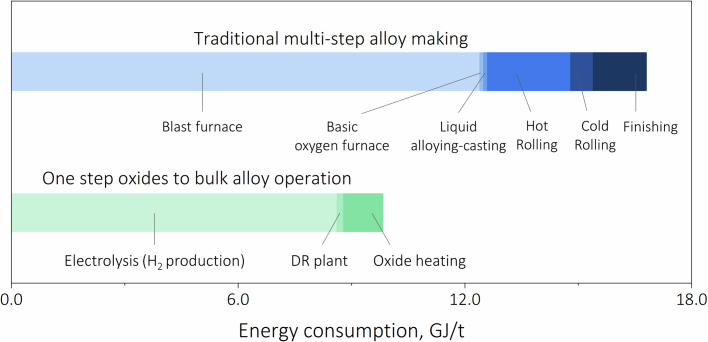
Rough estimation of the approximate energy consumption between the traditional multi-step alloy making approach and the proposed one step oxides to bulk alloy operation. While quantitatively precise cost comparison between these two methods still requires dedicated technoeconomic modelling, the major energy consumption might still be estimated based on the available dataset in the literature^63,64^. The estimated total energy consumption of the proposed *oxides to bulk alloy* synthesis method is ~9.83 GJ/t, significantly lower than the energy cost required by the conventional metal extraction-liquid alloying-thermomechanical processing route (~16.8 GJ/t).

To explore the phase transition details and the substitutional alloying process involved in the reduction, in situ synchrotron X-ray measurements were carried out at beamline P02.1, PETRA III of DESY. Similar to the ex situ measurements detailed above, a high-energy X-ray beam with a wavelength of 0.20735 Å was used. The working distance was chosen as 1,700 mm (with fine calibration using the NIST standard LaB_6_ powders), which ensures an optimal balance between the angular resolution and the number of Debye–Scherrer rings. The pre-compacted Fe_2_O_3_ and NiO powders were sealed inside a fused silica glass capillary tube with an inner diameter of about 0.6 mm and a standard type-K thermocouple was installed right next to the powders for temperature measurement. The capillary tube and the specimen were altogether heated up using a hot air blower equipped with a proportional–integral–derivative controller. Temperature calibration was also carried out to account for the potential deviation between the set temperature for the hot air blower and the actual specimen temperature. H_2_ gas of 1 atom purity was supplied through the capillarity tube during heating and a moderate flow rate of around 10 ml min^−1^ was adopted to mitigate convection cooling. A schematic of the experimental set-up is shown in Fig. 3a and its actual configuration is provided in Extended Data Fig. 5. During the experiment, two-dimensional diffractograms were acquired every 2 s (beam size 500 × 500 μm^2^) to closely monitor the inception of reduction and phase transitions involved. The lattice thermal expansion of the synthesized invar alloys (detailed in the preceding two sections) was assessed using the same experimental set-up, although the specimen was instead heated up in an Ar atmosphere. Post-analysis of the raw diffractograms was done using a GSAS-II open access software^49^ in which azimuthal integration was performed using a quarter (azimuthal angle 0°–90°) of the Debye–Scherrer ring. Diffraction peak shift was analysed using a Gaussian–Lorentzian peak-fitting algorithm^50^ and phase constitution evolution was further quantified by Rietveld refinement and a residual error weighted-profile *R*-factor *R*_wp_ < 10% was ensured for each diffractogram.

## Online content

Any methods, additional references, Nature Portfolio reporting summaries, source data, extended data, supplementary information, acknowledgements, peer review information; details of author contributions and competing interests; and statements of data and code availability are available at 10.1038/s41586-024-07932-w.

## Supplementary information

Supplementary Video 1 In situ synchrotron X-ray observations of invar alloy synthesis from Fe_2_O_3_ and NiO.

## Data Availability

The data that support the findings of this study are available from the corresponding author upon reasonable request.
